# Validating hierarchical verbal autopsy expert algorithms in a large data set with known causes of death

**DOI:** 10.7189/jogh.06.010601

**Published:** 2016-06

**Authors:** Henry D Kalter, Jamie Perin, Robert E Black

**Affiliations:** 1 Health Systems Program, Department of International Health, Johns Hopkins Bloomberg School of Public Health, Baltimore, MD, USA; 2 Center for Child and Community Health Research, Department of Pediatrics, Johns Hopkins School of Medicine, Baltimore, MD, USA; 3 Institute for International Programs, Department of International Health, Johns Hopkins Bloomberg School of Public Health, Baltimore, MD, USA

## Abstract

**Background:**

Physician assessment historically has been the most common method of analyzing verbal autopsy (VA) data. Recently, the World Health Organization endorsed two automated methods, Tariff 2.0 and InterVA–4, which promise greater objectivity and lower cost. A disadvantage of the Tariff method is that it requires a training data set from a prior validation study, while InterVA relies on clinically specified conditional probabilities. We undertook to validate the hierarchical expert algorithm analysis of VA data, an automated, intuitive, deterministic method that does not require a training data set.

**Methods:**

Using Population Health Metrics Research Consortium study hospital source data, we compared the primary causes of 1629 neonatal and 1456 1–59 month–old child deaths from VA expert algorithms arranged in a hierarchy to their reference standard causes. The expert algorithms were held constant, while five prior and one new “compromise” neonatal hierarchy, and three former child hierarchies were tested. For each comparison, the reference standard data were resampled 1000 times within the range of cause–specific mortality fractions (CSMF) for one of three approximated community scenarios in the 2013 WHO global causes of death, plus one random mortality cause proportions scenario. We utilized CSMF accuracy to assess overall population–level validity, and the absolute difference between VA and reference standard CSMFs to examine particular causes. Chance–corrected concordance (CCC) and Cohen’s kappa were used to evaluate individual–level cause assignment.

**Results:**

Overall CSMF accuracy for the best–performing expert algorithm hierarchy was 0.80 (range 0.57–0.96) for neonatal deaths and 0.76 (0.50–0.97) for child deaths. Performance for particular causes of death varied, with fairly flat estimated CSMF over a range of reference values for several causes. Performance at the individual diagnosis level was also less favorable than that for overall CSMF (neonatal: best CCC = 0.23, range 0.16–0.33; best kappa = 0.29, 0.23–0.35; child: best CCC = 0.40, 0.19–0.45; best kappa = 0.29, 0.07–0.35).

**Conclusions:**

Expert algorithms in a hierarchy offer an accessible, automated method for assigning VA causes of death. Overall population–level accuracy is similar to that of more complex machine learning methods, but without need for a training data set from a prior validation study.

For decades, health officials and program managers in low and middle income countries (LMIC) without well–functioning vital registration systems have used information on causes of death from verbal autopsy (VA) to allocate scarce resources to target the most common causes of child death. Simultaneously, the World Health Organization (WHO) and UNICEF, through their Child Health Epidemiology Reference Group (CHERG), have used VA data from the world’s public health literature to model and track the causes of neonatal and child death in LMIC countries [1–4]. However, VA data collection and analysis methods, including those of studies that have contributed input data to the CHERG models, have suffered from a lack of standardization and uncertainty as to the accuracy of their cause of death findings [5].

Until lately most studies have relied on physician analysis of VA findings, which has raised questions regarding the potential introduction of subjectivity and cultural biases into the VA diagnoses, as well as the monetary and health system costs of diverting physicians from patient care to the task of VA analysis [6]. Expert algorithms also have been used for VA analysis, with validation studies demonstrating fair to good accuracy for the diagnosis of several causes of neonatal and child death [7–10]; but this method has more often been used in research settings, with program environments being more comfortable with physician analysis. More recently, several machine learning and probabilistic VA analysis methods have been developed that show promise for providing more accurate diagnoses, as well as the objectivity that comes with automated methods and the efficiency and cost savings of not requiring physicians to conduct the analysis [11]. WHO recently modified its standardized VA questionnaire for use with two of these automated methods, Tariff 2.0 [12] and InterVA–4 [13], and is encouraging the use of these methods instead of the traditional physician review method [14].

However, questions remain as to which method or methods is most accurate, with a recent assessment emphasizing that different methods may work best for different age groups and causes of death [15]. Lastly, none of these studies examined the use of expert algorithms arranged in a hierarchy to select the primary cause of death, which offers the same advantages as other automated methods plus the additional benefit, unlike the Tariff method, of not requiring a training data set from a prior VA validation study, preferably conducted in the same geographic region or disease setting intended for the use of verbal autopsy, and distinct from all other automated methods, is based on clinical algorithms that can be easily explained to non–medical professionals. A later study did examine the performance of hierarchical algorithms, but in a small data set against physician–determined reference standard diagnoses using algorithms refined by physicians at the same sites, and missing some key neonatal causes of death [16]. Therefore, we undertook to validate the hierarchical expert algorithm VA analysis method in a large data set with objective reference standard criteria for a full range of important neonatal and child causes of death, and report the findings of our analyses in this paper.

## METHODS

We used source data from the Population Health Metrics Research Consortium (PHMRC) study to validate causes of under–five year–old deaths from verbal autopsy expert algorithms arranged in a hierarchy compared to reference standard causes of death. The design and primary results of the PHMRC study have been described in detail [17]. In brief, the study identified hospital deaths of all ages, including 1629 neonatal deaths and 1456 1–59 months old child deaths, at six study sites in five countries on three continents, determined the main or underlying reference standard cause for each death from available clinical, laboratory and imaging data, and later visited the household of each decedent to conduct a verbal autopsy interview. A large portion of these data are publicly available [18], although some questions about its contents have risen from the verbal autopsy research community [19]. For this reason, we conducted extensive cleaning of the PHMRC data to make it more suitable for our expert algorithm analysis, and have provided the cleaned data, documentation and cleaning information online [20]). We excluded stillbirths and deaths of persons older than five years from our analysis, restricting our interest to deaths of live born children who died before age five, analyzed separately for neonates 0 to 27 days and children 1 to 59 months old.

### Verbal autopsy cause of death assignment

Verbal autopsy (VA) expert algorithms are combinations of illness signs and symptoms judged by verbal autopsy researchers to be predictive of particular causes of death. The algorithms validated in the current study were based on those developed by researchers for prior VA validation studies, further consultation with additional verbal autopsy experts, and a literature review to identify illness signs and symptoms commonly associated with particular neonatal and child illnesses. The sources and algorithms themselves are provided in a recent publication [21]. We used the expert algorithms to estimate cause of death given each individual’s PHMRC VA questionnaire responses. While the PHMRC questionnaire includes close–ended questions on illness signs and symptoms, an open–ended narrative response and recording of data from medical records and death certificates available in the home, the expert algorithms are based only on the responses to close–ended questions on illness signs and symptoms.

Because the algorithms determine all contributing causes, in the event that more than one cause was identified the primary cause was chosen according to a pre–specified hierarchy. We determined the primary causes of neonatal death utilizing the same algorithms across five hierarchies for neonatal deaths that are currently in use: Arifeen et al. [22], Baqui et al. [23], Kalter et al. [21], Lawn et al. [24], and Liu et al. [25]; and the primary causes of child death (1–59 months of age) utilizing the hierarchies for this age group described by Arifeen et al. [22], Kalter et al. [21], and Liu et al. [25]. Other things being equal, estimating more causes at once will yield lower accuracy than estimating fewer causes [26]. Therefore, for neonatal deaths, we also examined a compromise hierarchy that included four cause categories in common across all five neonatal hierarchies (**Table 1**).

**Table 1:** Cause assignment hierarchies for determining the main cause of death among co–morbid causes in neonates 0–27 days and 1–59 month–old children

Arifeen et al. 2004 [22]	Baqui et al. 2006 [23]	Kalter et al. 2015 [21]	Lawn et al. 2006 [24]	Liu et al. 2015 [25]	Compromise
**Neonates 0–27 days:**					
Neonatal tetanus	Neonatal tetanus, Congenital abnormality	Neonatal tetanus	Congenital abnormality	Neonatal tetanus	Congenital abnormality
Congenital abnormality	Preterm delivery	Congenital abnormality	Neonatal tetanus	Congenital abnormality	Birth asphyxia
Birth asphyxia	Birth asphyxia	Birth asphyxia, birth injury	Preterm birth	Birth asphyxia, birth injury	Prematurity
Birth injury	Birth injury	Meningitis	Birth asphyxia	Diarrhea, ARI	Sepsis, pneumonia, meningitis
ARI, diarrhea	Sepsis or pneumonia	Diarrhea	Sepsis, pneumonia, meningitis	Meningitis	
Possible diarrhea, possible ARI, sepsis	Diarrhea	Pneumonia	Diarrhea	Possible pneumonia, possible diarrhea	
Premature birth/LBW	Unspecified	Possible diarrhea	Other	Prematurity/LBW	
Other causes		Possible pneumonia		Sepsis, other possible serious infections	
Unspecified		Sepsis		Unspecified	
		Jaundice			
		Hemorrhagic disease of the newborn			
		Sudden unexplained death			
		Preterm delivery			
		Unspecified			
**Children 1–59 months:**					
Injury		Injury		Injury	
ARI, diarrhea, measles		AIDS		Measles, diarrhea, ARI	
Possible serious infections		Malnutrition (underlying)		Meningitis	
Malnutrition		Measles		Malaria	
Other causes		Meningitis		AIDS	
Unspecified		Dysentery		Possible diarrhea/ARI	
Undetermined		Diarrhea		Other possible serious infections	
		Pertussis		Unspecified	
		Pneumonia			
		Malaria			
		Possible dysentery			
		Possible diarrhea			
		Possible pneumonia			
		Hemorrhagic fever			
		Other infection			
		Residual infection			
		Malnutrition			
		Unspecified			

LBW – low birth weight, ARI – acute respiratory infection

### Reference standard cause of death

We used the reference standard causes of death from the PHMRC study to approximate the cause of death distribution in community settings, where verbal autopsy is most relevant. Because the PHMRC study was hospital– as opposed to community based, and the cause distribution in the community and hospital may differ, we resampled from the study deaths to represent a variety of cause distributions.

We approximated three specific mortality settings with the PHMRC data: (1) communities with high under five mortality where malaria is endemic, (2) communities with high under five mortality where malaria is not endemic, and (3) communities with moderate under five mortality. We used the Child Health Epidemiology Reference Group (CHERG) definition of high under–five mortality (more than 35 deaths per 1000 live births) [4], took moderate mortality as 20 to 35 deaths per 1000, and defined malaria endemicity as greater than 5 percent of under–five deaths due to malaria. In addition to these three specific scenarios of interest, we also considered a fourth general scenario, where all cause-specific mortality fractions were randomly varied between 5% and 40%.

Estimated cause proportions of death for all countries in the world, including those where most deaths occur outside the formal health sector, are available from the WHO [4]. We used these estimated causes of neonatal and child mortality as a guide in choosing cause distributions in our scenarios of interest. To generate a possible set of verbal autopsies to represent a given death distribution in a particular mortality scenario, we selected one country at random among all those appropriate, and resampled the PHMRC questionnaire data to correspond approximately to that cause of death distribution. For neonates, we included deaths due to prematurity, birth asphyxia, congenital malformations, meningitis, pneumonia and sepsis; and for children we used deaths from HIV, diarrhea, measles, meningitis/encephalitis, malaria, pneumonia, injuries, other infectious causes, and non–infectious causes. Some causes of interest for Liu et al. [4] do not occur in the PHMRC study data, requiring that we use relative proportions of causes reported by the PHMRC, while unreported causes were not considered. For example, the tetanus mortality fraction for neonatal deaths as reported by WHO is as high as 8%, but there are no neonatal deaths due to tetanus in the PHMRC data.

The PHMRC data include neonatal deaths due to co–morbid preterm delivery, birth asphyxia and/or sepsis; and child deaths due to co–morbid pneumonia and diarrhea. For deaths with co–morbid reference standard causes of death, we used the ICD–10 rules to assign a single underlying cause of death [27]. In accordance with the rule that the mode of perinatal death, including prematurity, should not be classified as the main disease or condition unless it was the only condition known, we assigned deaths due to co–morbid preterm/birth asphyxia to birth asphyxia, preterm/sepsis to sepsis, and preterm/sepsis/birth asphyxia proportionately to sepsis and birth asphyxia. Deaths from conditions directly due to prematurity, such as Respiratory Distress Syndrome, were classified as being due to preterm delivery. For child deaths, we proportionately reallocated co–morbid pneumonia/diarrhea deaths to pneumonia or diarrhea. Using these verbal autopsies for harmonized causes of death, we repeated our selection of cause of death distribution and resampling 1000 times for each of the four scenarios. **Table 2** summarizes our harmonization of the verbal autopsy algorithms and reference standard causes of death.

**Table 2:** Correspondence of verbal autopsy and reference standard diagnoses in the hierarchies

Verbal autopsy algorithm(s)	PHMRC reference standard group(s)	Placement in hierarchy
**Neonates 0–27 days:**		
Neonatal tetanus	No PHMRC neonatal tetanus cases	–
Congenital malformation	Congenital malformation	Malformation
Birth injury	No PHMRC birth injury cases	–
Birth asphyxia	Birth asphyxia, preterm delivery (without RDS) and birth asphyxia, preterm delivery (without RDS) and sepsis and birth asphyxia (allocated to birth asphyxia according to the distribution of other deaths due to sepsis and birth asphyxia)	Birth asphyxia
Meningitis	Meningitis (serious infection)	Meningitis
Diarrhea	No PHMRC neonatal diarrhea cases	–
Pneumonia; ARI	Pneumonia (serious infection)	Pneumonia
Possible diarrhea	No PHMRC neonatal diarrhea cases	–
Possible pneumonia, possible ARI	Pneumonia (serious infection)	Possible pneumonia (later to combine with pneumonia)
Sepsis	Sepsis (serious infection), sepsis with local bacterial infection, preterm delivery (with or without RDS) and sepsis, preterm delivery (without RDS) and sepsis and birth asphyxia (allocated to sepsis according to the distribution of other deaths due to sepsis and birth asphyxia)	Sepsis
Jaundice	No PHMRC jaundice cases	–
Hemorrhagic disease of the newborn	No PHMRC hemorrhagic disease of the newborn cases	–
Sudden unexplained death	No PHMRC sudden unexplained death cases	–
Preterm delivery, Preterm delivery with complication specific to prematurity (RDS)	Preterm delivery (<33 weeks gestational age [GA]) with or without RDS, preterm delivery (33–36 weeks GA) with RDS	Preterm delivery
**Children 1–59 months:**		
Injury	Bite of a venomous animal, burn, drowning, fall, poisoning, road traffic injury, violent death	Injury
AIDS	AIDS	AIDS
Malnutrition (underlying)	No PHMRC malnutrition cases	–
Measles	Measles	Measles
Meningitis	Encephalitis, meningitis	Meningitis
Diarrhea or dysentery	Diarrhea/dysentery	Diarrhea/dysentery
Pneumonia or diarrhea	Pneumonia and diarrhea	Allocated to pneumonia and diarrhea/dysentery according to the distribution of other deaths due to pneumonia and diarrhea/dysentery
Pneumonia	Pneumonia	Pneumonia
Malaria	Malaria	Malaria
Possible diarrhea or dysentery	Diarrhea/dysentery	Possible diarrhea or dysentery (later to combine with diarrhea/dysentery)
Possible pneumonia	Pneumonia	Possible pneumonia (later to combine with pneumonia)
Pertussis, hemorrhagic fever, other infection	Hemorrhagic fever, sepsis, tuberculosis, other infectious diseases	Other infectious causes
Residual infection (possible malaria)	Malaria	Possible malaria (later to combine with malaria)

PHMRC – Population Health Metric Research Consortium, RDS – respiratory distress syndrome, ARI – acute respiratory infection

### Accuracy of VA cause of death determination

After resampling the reference standard cause of death data for neonates and children according to the four mortality scenarios as described above, we then, separately for neonatal and child deaths and for each hierarchy in each scenario, used the expert algorithms to estimate cause of death in the resampled reference standard cause of death data given each individual’s VA questionnaire responses. We used four metrics to examine the validity of the VA cause of death estimates, two at the population level and two at the level of individual cause assignment. Cause-specific mortality fraction (CSMF) accuracy, as defined by Murray et al. [28], is an overall summary of the estimated and reference standard cause distributions with larger values indicating VA CSMF measurements closer to the reference standard. CSMF accuracy is the sum of absolute errors by cause, scaled by the extent of possible error given the smallest cause fraction, and subtracted from one. It is generally interpretable as percent accuracy. To assess the validity of VA estimates of particular causes of death we examined the absolute difference between VA and reference standard CSMFs for these causes.

The last two metrics estimate the accuracy of VA cause of death assignment at the level of individual deaths. Cohen’s kappa is a general measure of agreement between estimated and reference standard causes [29]. Large values of kappa indicate more agreement, where in general values less than zero indicate no agreement, values between 0 and 0.2 are rated as minimal agreement, 0.2 to 0.4 as fair, 0.4 to 0.6 as moderate, 0.6 to 0.8 as substantial, and 0.8 to 1 approach exact agreement [30]. Chance corrected concordance (CCC) is another measure of agreement between VA and reference standard causes at the individual level. This statistic is closely related to Cohen’s kappa and average sensitivity across causes or categories [28]. Similar to kappa, large values indicate more agreement. The CCC scale is from 1/(1–N) to 1, for the number of causes N, while the scale for Cohen’s kappa is from –1 to 1. We used these two metrics only to generate overall summaries of VA accuracy for all causes together.

### Ethics statement

The study data are publically accessible and include no personal identifiers. Therefore, no ethical review of the study protocol or informed consent was necessary.

## RESULTS

### Neonates

**Figure 1:**
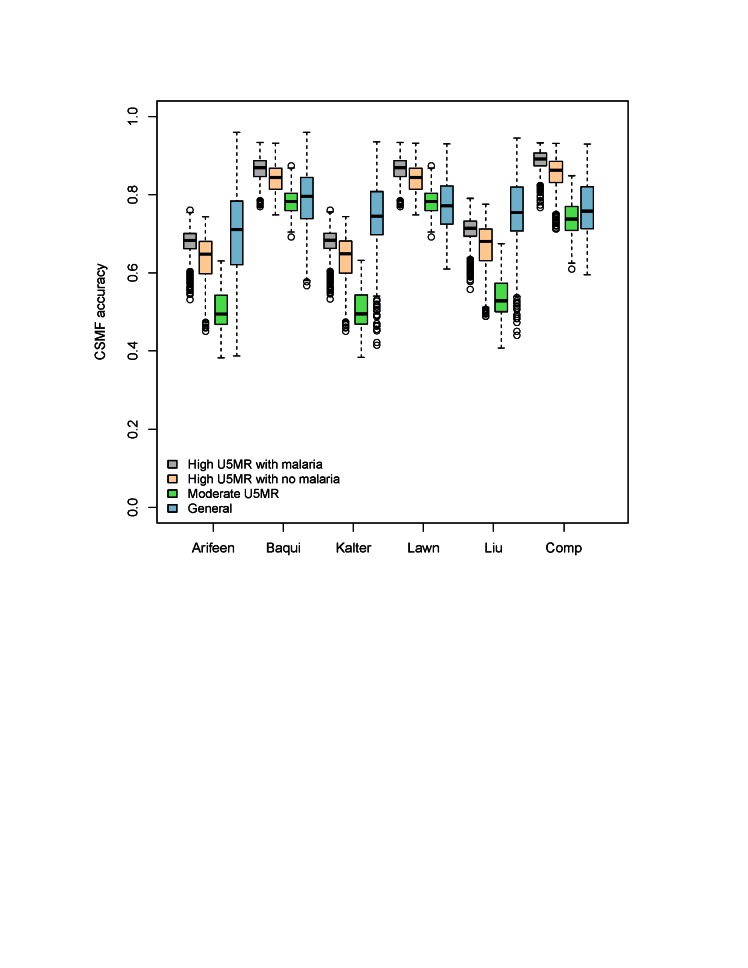
Cause-specific mortality fraction accuracy for six neonatal expert algorithm hierarchies in the resampled Population Health Metrics Research Consortium data, for 1000 simulated cause distributions from four neonatal mortality scenarios, and four neonatal causes (birth asphyxia, congenital malformation, prematurity, sepsis/pneumonia or sepsis/pneumonia/meningitis). Boxes represent interquartile ranges, with a line at the median. Whiskers represent 95% confidence intervals for the median values, and outliers are shown by dots.

**Table 3** shows summary results for the expert algorithm cause of death assignments for all causes together from four mortality scenarios and three measures of accuracy. By the CSMF measure, the Baqui and Lawn hierarchies performed best in the moderate and general mortality scenarios, and the compromise hierarchy did best in both high mortality scenarios. These three hierarchies all did their best in the high mortality scenarios, whereas the Kalter and Liu hierarchies did their best in the general scenario, in which their performance nearly equaled that of the Lawn hierarchy. All the hierarchies did their worst, or nearly so, in the moderate mortality scenario. **Figure 1** also summarizes CSMF accuracy for neonatal deaths in these scenarios.

**Table 3:** Agreement* of reference standard and algorithm cause of death assignment among neonates

Scenario	Arifeen et al. 2004 [22]	Baqui et al. 2006 [23]	Kalter et al. 2015 [21]	Lawn et al. 2006 [24]	Liu et al. 2015 [25]	Compromise
**Cause-specific mortality fraction accuracy:**						
High U5MR with malaria	0.68 (0.53–0.76)	0.87 (0.77–0.93)	0.68 (0.53–0.76)	0.87 (0.77–0.93)	0.71 (0.56–0.79)	0.89 (0.77–0.93)
High U5MR without malaria	0.65 (0.45–0.74)	0.84 (0.75–0.93)	0.65 (0.45–0.74)	0.84 (0.75–0.93)	0.68 (0.49–0.78)	0.86 (0.71–0.93)
Moderate U5MR	0.49 (0.38–0.63)	0.78 (0.69–0.87)	0.49 (0.38–0.63)	0.78 (0.69–0.87)	0.53 (0.41–0.67)	0.74 (0.61–0.85)
General	0.71 (0.39–0.96)	0.80 (0.57–0.96)	0.74 (0.41–0.94)	0.77 (0.61–0.93)	0.75 (0.44–0.95)	0.76 (0.60–0.93)
**Cohen’s kappa:**						
High U5MR with malaria	0.17 (0.12–0.22)	0.29 (0.23–0.35)	0.17 (0.12–0.22)	0.29 (0.23–0.35)	0.18 (0.13–0.23)	0.26 (0.21–0.31)
High U5MR without malaria	0.17 (0.11–0.22)	0.29 (0.24–0.36)	0.17 (0.11–0.22)	0.29 (0.24–0.36)	0.18 (0.12–0.22)	0.26 (0.21–0.32)
Moderate U5MR	0.15 (0.04–0.21)	0.28 (0.16–0.34)	0.15 (0.04–0.21)	0.28 (0.16–0.34)	0.16 (0.04–0.21)	0.24 (0.11–0.29)
General	0.14 (0.07–0.23)	0.20 (0.08–0.36)	0.15 (0.06–0.24)	0.24 (0.12–0.37)	0.16 (0.07–0.25)	0.22 (0.11–0.33)
**Chance corrected concordance:**						
High U5MR with malaria	0.13 (0.06–0.19)	0.22 (0.16–0.28)	0.13 (0.06–0.19)	0.22 (0.16–0.28)	0.14 (0.07–0.20)	0.20 (0.14–0.26)
High U5MR without malaria	0.13 (0.09–0.18)	0.22 (0.17–0.28)	0.13 (0.09–0.18)	0.22 (0.17–0.28)	0.14 (0.09–0.19)	0.20 (0.15–0.26)
Moderate U5MR	0.13 (0.09–0.22)	0.22 (0.17–0.44)	0.13 (0.09–0.22)	0.22 (0.17–0.44)	0.14 (0.09–0.23)	0.20 (0.15–0.36)
General	0.14 (0.08–0.22)	0.23 (0.16–0.33)	0.12 (0.05–0.21)	0.23 (0.17–0.32)	0.13 (0.06–0.22)	0.21 (0.14–0.30)

U5MR – under 5 years mortality rate

*Median and range across 1000 simulated instances of the Population Health Metrics Research Consortium study data for cause-specific mortality fraction (CSMF) accuracy, the kappa statistic, and chance corrected concordance (CCC) by mortality scenario and hierarchical method for distributing co–morbid causes of neonatal death, for four causes: birth asphyxia, congenital malformation, prematurity, sepsis/pneumonia or sepsis/pneumonia/meningitis.

The Baqui and Lawn hierarchies performed best by the Cohen’s kappa measure, followed closely by the compromise hierarchy. Generally, for all algorithms, the Cohen’s kappa was between 0.1 and 0.4, indicating minimal to fair agreement between VA estimated and reference standard causes. The CCC statistic also indicates that expert algorithms in the Baqui and Lawn hierarchies provide estimates that are closer to the reference standard causes than either the Kalter or Liu hierarchies, but overall the CCC statistics for all the hierarchies are between 0 and 0.45, indicating small to moderate agreement with the reference standard causes.

**Figure 2:**
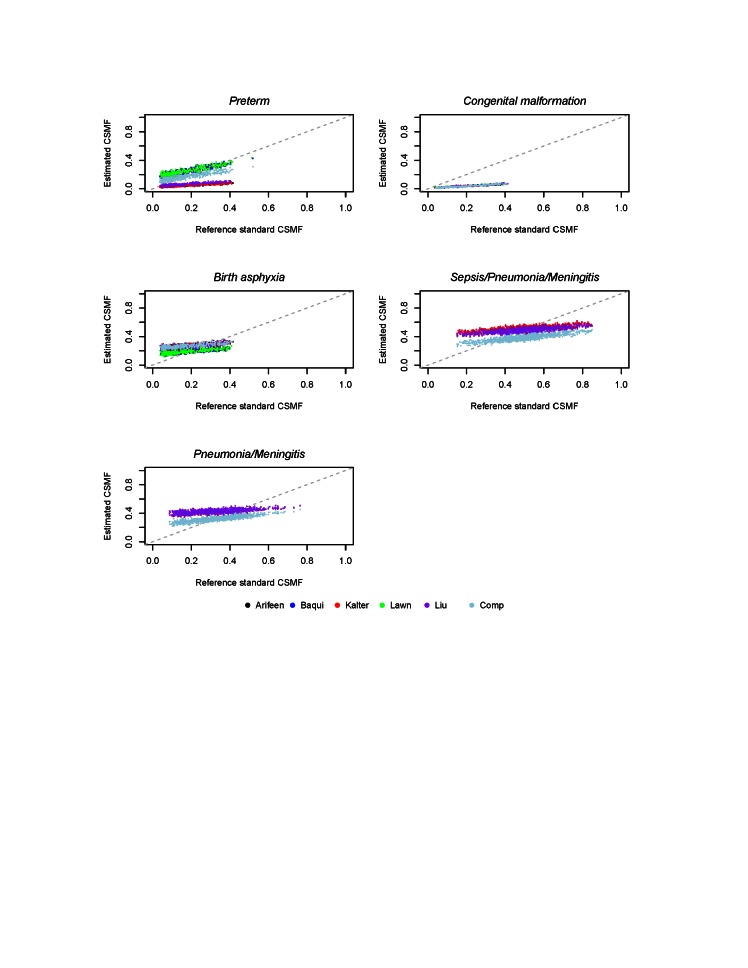
Cause-specific mortality fractions for six neonatal expert algorithm hierarchies in the resampled Population Health Metrics Research Consortium data, for four neonatal causes in the general neonatal mortality scenario, for 1000 simulated cause distributions.

The median and range of absolute differences between estimated and reference standard CSMFs are shown in **Table 4** for each neonatal cause of death, along with the proportion of deaths that were not classified by each hierarchy. **Figure 2** shows the simulated reference standard and estimated CSMF in the general mortality scenario. This difference is identical across all hierarchies for the percent of deaths due to congenital malformations, because this cause is the first in each hierarchy. The Baqui and Lawn hierarchies perform best for birth asphyxia, and Baqui is best for sepsis/pneumonia. The compromise hierarchy is best for prematurity, and the Lawn and compromise hierarchies are jointly best for sepsis/meningitis/pneumonia.

**Table 4:** Absolute difference* between the cause-specific mortality fraction of each estimated and reference standard cause, for the general neonatal mortality scenario

Cause	Arifeen et al. 2004 [22]	Baqui et al. 2006 [23]	Kalter et al. 2015 [21]	Lawn et al. 2006 [24]	Liu et al. 2015 [25]	Compromise
Birth asphyxia	0.11 (0.00–0.22)	0.07 (0.01–0.15)	0.11 (0.00–0.22)	0.07 (0.01–0.15)	0.11 (0.00–0.22)	0.11 (0.00–0.22)
Congenital malformation	0.13 (0.03–0.31)	0.13 (0.03–0.31)	0.13 (0.03–0.31)	0.13 (0.03–0.31)	0.13 (0.03–0.31)	0.13 (0.03–0.31)
Meningitis	–	–	0.09 (0.01–0.24)	–	0.10 (0.01–0.24)	–
Pneumonia	–	–	0.19 (0.01–0.32)		0.20 (0.01–0.32)	–
Prematurity	0.12 (0.02–0.30)	0.08 (0.00–0.17)	0.12 (0.02–0.30)	0.08 (0.00–0.17)	0.11 (0.00–0.28)	0.05 (0.00–0.13)
Sepsis	–	–	0.09 (0.00–0.28)		0.11 (0.00–0.30)	0.11 (0.00–0.30)
Sepsis/pneumonia	0.18 (0.02–0.36)	0.10 (0.00–0.24)	0.12 (0.01–0.29)		0.11 (0.00–0.27)	–
Sepsis/pneumonia/meningitis	–	–	0.22 (0.18–0.30)	0.03 (0.00–0.08)	0.19 (0.15–0.27)	0.03 (0.00–0.08)
Unspecified	0.15 (0.12– 0.19)	0.15 (0.12– 0.19)	0.14 (0.11–0.18)	0.14 (0.11–0.18)	0.14 (0.11–0.18)	0.14 (0.11–0.18)

*Median and range across one thousand simulations. Results are shown for six hierarchies as a proportion of all neonatal deaths.

### Children

**Figure 3:**
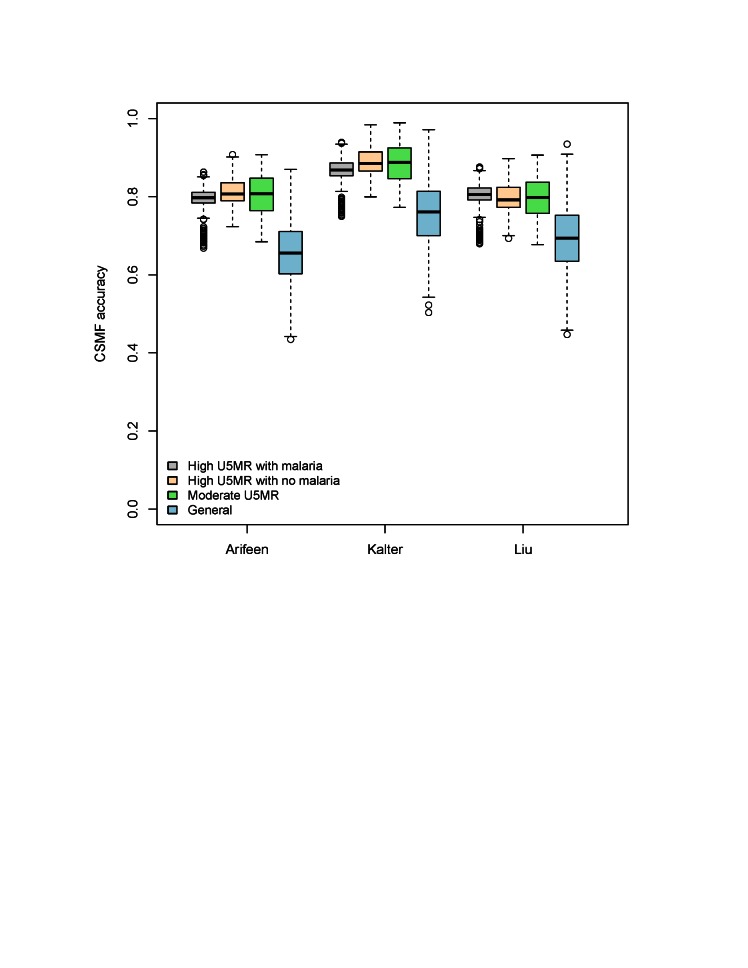
Cause-specific mortality fraction accuracy for three expert algorithm hierarchies in the resampled Population Health Metrics Research Consortium data, for 1000 simulated cause distributions from four neonatal mortality scenarios, and four causes of child death (pneumonia/diarrhea, measles, other infectious causes, and injury). Boxes represent interquartile ranges, with a line at the median. Whiskers represent 95% confidence intervals for the median values, and outliers are shown by dots.

**Table 5** shows summary results for the expert algorithm cause assignment of four causes of child deaths in three hierarchies and four mortality scenarios. We used the same three measures of accuracy as for neonatal deaths at the population and individual levels. At the population level, summarized by CSMF accuracy, the Kalter hierarchy performs best in each scenario. This population level comparison is also shown in **Figure 3**.

**Table 5:** Agreement* of reference standard and algorithm cause of death assignment among children 1–59 months old

Scenario	Arifeen et al. 2004 [22]	Kalter et al. 2015 [21]	Liu et al. 2015 [25]
**Cause-specific mortality fraction accuracy:**			
High U5MR with malaria	0.80 (0.67–0.86)	0.87 (0.75–0.94)	0.81 (0.68–0.88)
High U5MR without malaria	0.83 (0.73–0.90)	0.93 (0.79–0.97)	0.80 (0.69–0.90)
Moderate U5MR	0.84 (0.74–0.90)	0.92 (0.79–0.97)	0.80 (0.68–0.91)
General	0.66 (0.43–0.87)	0.76 (0.50–0.97)	0.69 (0.45–0.93)
**Cohen’s kappa:**			
High U5MR with malaria	0.14 (0.06–0.25)	0.13 (0.07–0.22)	0.14 (0.08–0.22)
High U5MR without malaria	0.24 (0.09–0.35)	0.21 (0.08–0.32)	0.23 (0.09–0.34)
Moderate U5MR	0.29 (0.07–0.35)	0.25 (0.08–0.32)	0.28 (0.08–0.35)
General	0.10 (0.02–0.38)	0.10 (0.04–0.33)	0.10 (0.04–0.35)
**Chance corrected concordance:**			
High U5MR with malaria	0.25 (0.20–0.49)	0.17 (0.12–0.39)	0.20 (0.14–0.42)
High U5MR without malaria	0.23 (0.18–0.45)	0.22 (0.17–0.46)	0.22 (0.18–0.44)
Moderate U5MR	0.40 (0.19–0.45)	0.37 (0.16–0.55)	0.39 (0.17–0.48)
General	0.24 (0.16–0.30)	0.16 (0.10–0.23)	0.19 (0.12–0.25)

U5MR – under 5 years mortality rate

*Median and range across 1000 simulated instances of the Population Health Metrics Research Consortium study data for cause-specific mortality fraction (CSMF) accuracy, the kappa statistic, and chance corrected concordance (CCC) by mortality scenario and hierarchical method for distributing co–morbid causes of child death, for four causes: pneumonia/diarrhea, measles, other infectious causes, and injury.

The hierarchies are not as strongly differentiated at the individual level for child deaths. There is also some counter indication at the individual level between Cohen’s kappa and the CCC statistic as to which hierarchy is best in each mortality scenario. By Cohen’s kappa, the three hierarchies are very similar in the high mortality with malaria and the general mortality scenarios. Also by Cohen’s kappa, the Liu and Arifeen hierarchies are similar in the high mortality without malaria and moderate mortality scenarios, while the Kalter hierarchy has somewhat lower agreement. The Cohen’s kappa for these three hierarchies generally range from slight (less than 0.2) to fair agreement (0.2–0.4).

By the CCC statistic, Arifeen’s hierarchy has the largest median across the mortality scenarios, although the advantage is small, especially for the high mortality without malaria and moderate mortality scenarios. Overall CCC statistics range from 0.06 to 0.55, indicating small to moderate agreement by the standards for interpreting Cohen’s kappa, and somewhat higher agreement than for neonates.

**Figure 4:**
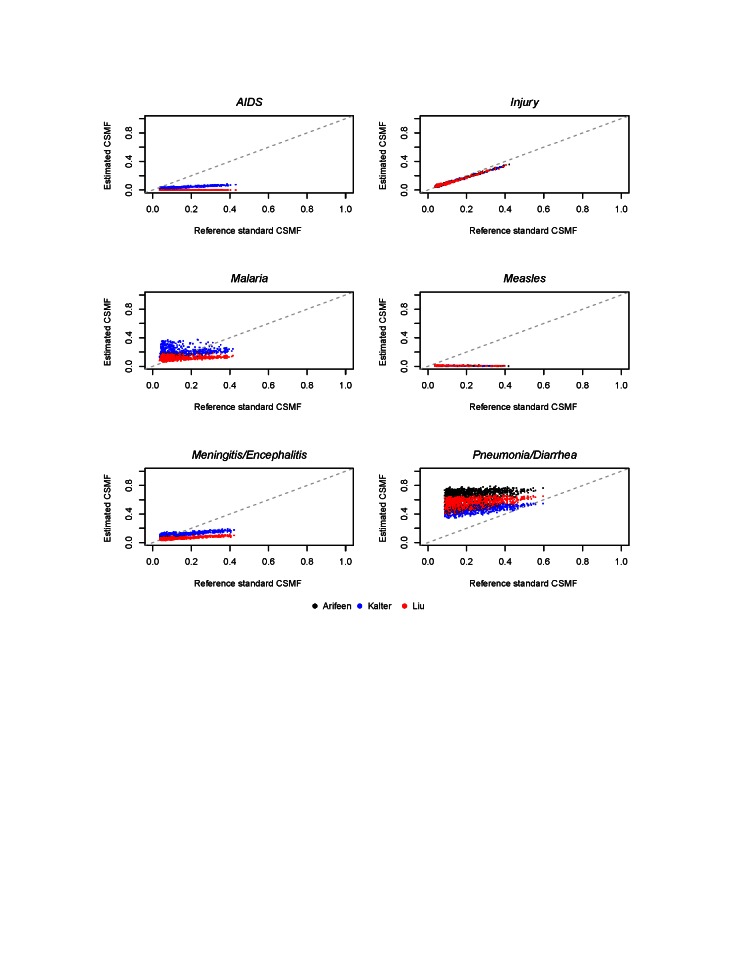
Cause-specific mortality fractions for three expert algorithm hierarchies in the resampled Population Health Metrics Research Consortium data, for six child causes in the general mortality scenario, for 1000 simulated cause distributions.

**Figure 4** shows the simulated reference standard and estimated CSMF in the general mortality scenario for six causes of child deaths. The median and range of absolute differences between estimated and reference standard CSMFs across these simulated instances of the PHMRC data for each cause of child death are shown in **Table 6**. This difference is identical across all hierarchies for the percent of deaths due to injuries, because this cause occupies the same place in the respective hierarchies. The Kalter hierarchy is best for pneumonia/diarrhea, meningitis/encephalitis, and AIDS. The Arifeen hierarchy is best for other infectious causes, while the Liu hierarchy is generally best for malaria. The Liu hierarchy is especially accurate when malaria is below 0.10 CSMF, while the Kalter hierarchy tends to be more accurate as malaria increases, as shown in **Figure 4**. **Table 6** shows the median absolute difference in CSMF, which may mask differences depending on the reference standard CSMF.

**Table 6:** Absolute difference* between the cause-specific mortality fraction of each estimated and reference standard cause, for the general, high mortality with malaria and high mortality without malaria child mortality scenarios

Cause	Arifeen et al. 2004 [22]	Kalter et al. 2015 [21]	Liu et al. 2015 [25]
**Child – General mortality scenario:**			
AIDS	–	0.08 (0.01–0.28)	0.08 (0.04–0.35)
Diarrhea/dysentery	–	0.08 (0.01–0.15)	–
Injury	0.01 (0.00–0.05)	0.01 (0.00–0.05)	0.01 (0.00–0.05)
Malaria	–	0.10 (0.01–0.26)	0.04 (0.00–0.22)
Measles	0.10 (0.03–0.36)	0.10 (0.03–0.36)	0.10 (0.03–0.36)
Meningitis/encephalitis	–	0.05 (0.00–0.20)	0.06 (0.00–0.27)
Other infectious causes	0.07 (0.00–0.25)	0.08 (0.00–0.32)	0.11 (0.04– 0.35)
Pneumonia	–	0.20 (0.03–0.30)	–
Pneumonia/diarrhea	0.46 (0.24–0.62)	0.26 (0.04–0.40)	0.37 (0.13–0.51)
Unspecified	0.47 (0.50– 0.95)	0.11 (0.00– 0.51)	0.14 (0.00–0.63)
**Child – High mortality with malaria:**			
AIDS	–	0.01 (0.00–0.10)	0.02 (0.00–0.13)
Diarrhea/dysentery	–	0.03 (0.00–0.08)	–
Injury	0.01 (0.00–0.01)	0.01 (0.00–0.01)	0.01 (0.00–0.01)
Malaria	–	0.09 (0.00–0.23)	0.15 (0.01–0.30)
Measles	0.01 (0.00–0.08)	0.01 (0.00–0.08)	0.01 (0.00–0.08)
Meningitis/encephalitis	–	0.08 (0.05–0.10)	0.02 (0.00–0.05)
Other infectious causes	0.03 (0.00–0.11)	0.09 (0.05–0.15)	0.12 (0.09–0.17)
Pneumonia	–	0.12 (0.06–0.23)	–
Pneumonia/diarrhea	0.35 (0.25–0.45)	0.14 (0.05–0.26)	0.24 (0.15–0.36)
Unspecified	0.45 (0.26–0.62)	0.06 (0.02–0.29)	0.10 (0.06–0.34)
**Child – High mortality without malaria:**			
AIDS	–	0.02 (0.00–0.30)	0.01 (0.00–0.37)
Diarrhea/dysentery	–	0.02 (0.00–0.09)	–
Injury	0.01 (0.00–0.02)	0.01 (0.00–0.02)	0.01 (0.00–0.02)
Malaria	–	–	–
Measles	0.02 (0.00–0.08)	0.02 (0.00–0.08)	0.02 (0.00–0.08)
Meningitis/encephalitis	–	0.06 (0.02–0.08)	0.14 (0.07–0.20)
Other infectious causes	0.03 (0.00–0.11)	0.09 (0.05–0.15)	0.12 (0.09–0.17)
Pneumonia	–	0.10 (0.06–0.16)	–
Pneumonia/diarrhea	0.22 (0.17–0.36)	0.09 (0.04–0.22)	0.19 (0.14–0.34)
Unspecified	0.26 (0.17–0.39)	0.10 (0.04–0.23)	0.16 (0.08–0.26)

*Median and range across one thousand simulations. Results are shown for three hierarchies, as a proportion of all child deaths.

The median absolute differences between estimated CSMF and reference CSMF by cause are also shown for the two high mortality scenarios in **Table 6**, both with and without malaria. The relative accuracy of the hierarchies by cause was similar to their performance in the general mortality scenario, except that in the high mortality with malaria scenario the Liu hierarchy did best for meningitis/encephalitis and the Kalter hierarchy worked best for malaria. The median absolute difference for pneumonia and diarrhea in the scenario for high mortality with malaria was 0.35, 0.14, and 0.24 for the Arifeen, Kalter, and Liu hierarchies respectively. These same median absolute differences in the high mortality scenario *without* malaria were 0.22, 0.09, and 0.19, indicating an improvement in estimated CSMF for pneumonia and diarrhea when the CSMF for deaths due to malaria was low. In addition, the median difference in the pneumonia CSMFs in the Kalter hierarchy was 0.12 in the high mortality scenario with malaria, and 0.10 in the high mortality scenario without malaria. These results reflect improved estimates for pneumonia, as expected given that high malaria burden may complicate other diagnoses, especially for pneumonia [31].

The software for the best performing neonatal and child algorithms and hierarchies, along with the PHMRC questionnaire needed to collect the input data, are available online [20].

## DISCUSSION

We have compared six expert algorithm hierarchies for assigning causes of neonatal death and three for assigning causes of child death, and we compared the resulting cause distributions with reference standard causes. We made these comparisons among the PHMRC study data, resampled to resemble the cause proportions of deaths from a variety of community settings as determined by the Child Health Epidemiology Reference Group on behalf of WHO. There was minimal to fair agreement between the algorithmic and the reference standard diagnoses at the individual level, both for neonatal and child causes of death, although some hierarchies had slightly higher agreement than others.

Verbal autopsies are generally used to describe populations instead of individuals, and so we have focused on measures of the agreement between algorithm–assigned and reference standard causes at the population level [32]. By this measure the agreement between assigned and reference standard causes was more favorable and the algorithms appear useful. When assessed in this manner, the Baqui, Lawn and compromise hierarchies performed best for neonatal causes, and the Kalter hierarchy performed best for children. The nearly equal performance of several hierarchies for neonatal deaths in the general mortality scenario suggests that several of the VA studies used as input data for the WHO/CHERG modeled estimates, whose cause distributions were the basis for the other mortality scenarios, may have used hierarchies with preterm placed higher up to select among multiple causes, similar to the ordering of diagnoses in the Baqui and Lawn hierarchies.

Hierarchy performance also varied across particular causes of neonatal and child death. For neonatal deaths, the Baqui and Lawn hierarchies performed best for birth asphyxia, and the compromise hierarchy performed best for prematurity. The Baqui hierarchy also performed best for sepsis/pneumonia, while the Lawn and compromise hierarchies performed best for sepsis/pneumonia/meningitis. For deaths in children 1–59 months, there was a striking difference in hierarchy performance for pneumonia, for which the Kalter hierarchy performed best. Clearly some causes are more difficult to classify than others. Hierarchy–estimated CSMF for child deaths due to injury was very close to the reference CSMF across all simulated scenarios. The estimated CSMF for measles, however, was near zero for all simulations, indicating a poor diagnostic ability, contrary to expectations for identifying measles [33]. This was likely due to an aberration in the PHMRC VA interview data, which identified ‘rash’ in only 3/23 reference standard measles cases [18].

Poor performance for particular causes may be masked by good overall performance as indicated by CSMF accuracy. For example, when an algorithm estimates 52%, 29%, 2% and 4% for neonatal deaths due to sepsis/pneumonia, birth asphyxia, congenital malformation, and prematurity, where the actual CSMFs are 32%, 31%, 11%, and 11%, the CSMF accuracy is 0.79, indicating good overall performance although sepsis/pneumonia is overestimated by 20%. Poor performance was observed for several causes in both neonates and children, where estimated CSMF was relatively flat over a range of reference standard CSMF. The CSMF accuracy as a statistic is limited in its ability to describe these details.

Until very recently the verbal autopsy standard was for questionnaires to be examined individually with cause of death determination by physician review. The new standard is to encourage assignment of cause of death using automated computer programs for the InterVA–4 and Tariff 2.0 methods [14]. The Tariff has been shown to outperform InterVA–4 in population level metrics, although reports vary [11,15]. The Tariff method determines cause based on the relative associations of symptoms and causes of death in a reference standard “training” data set, supplemented with global burden of disease estimates for questionnaires with undetermined cause of death [12].

In a validation study with the PHMRC data, CSMF accuracy of the Tariff 2.0 was reported at 0.81 (uncertainty 0.80, 0.82) for neonatal causes and 0.74 (uncertainty 0.74, 0.75) for child causes [12]. This is within the observed range of the best performing expert algorithm hierarchies (at 0.80 with range 0.57 to 0.96 for neonatal deaths and 0.76 with range 0.50 to 0.97 for child deaths), but with smaller uncertainty. The comparison, however, is not conclusive. Although the CSMF accuracy both of the expert algorithms and the Tariff were determined in the PHMRC data, only the Tariff was built on data from PHMRC study, potentially providing it with an advantage. In addition, the methods for resampling and estimating uncertainty were not the same, and so the reference is not necessarily on the same basis. In addition, the specified causes were not the same. For example, in the assessment of Tariff performance with neonatal causes of death, all deaths with co–morbid prematurity, birth asphyxia and/or sepsis were classified for resampling as being due to prematurity. The Tariff validation included six causes for neonatal deaths, and 21 for children, which is more total causes than in our expert algorithm validation. A definitive comparison of the Tariff and expert algorithm methods is further complicated by computational requirements of the Tariff. A single selection of deaths can be used to validate the expert algorithms, but in addition to these, the Tariff requires a selection of reference deaths for training. This comparison is outside the scope of this paper, but an area for further research.

The expert algorithms are fully deterministic: verbal autopsies with the same responses will be assigned the same cause of death. Algorithms are based on symptom patterns that physicians and medical experts expect to correspond to common causes of death in neonates and children. This determinism is an asset for facilitating use and understanding. While InterVA is also deterministic, it relies on conditional probabilities of the relationships between symptoms and causes of death that operate unseen in the background, rendering it less easily explainable to non–medical professionals. The Tariff, in contrast to both the VA algorithms and InterVA methods, requires a reference selection, from which symptom patterns are determined. The circumstances that require verbal autopsy are precisely where the cause of death distribution is unknown, precluding the selection of a perfect reference. The Tariff method’s sensitivity to this selection is not well understood, as a research friendly version has not been released. There is an unquantified potential for the Tariff to fail in the event that a poor reference is chosen.

This same determinism and predictability in the expert algorithm method that facilitates its use may be a liability in other respects. We observed some outlying cases of poor agreement between the predicted and reference standard cause fractions, although overall there was good agreement at the population level between algorithm and reference standard causes.

## CONCLUSION

Verbal autopsy is an invaluable tool in settings where civil registration is unreliable or incomplete. Health policy makers and programmers need verbal autopsy to better understand the causes of neonatal and child deaths and how these deaths might have been prevented. Here we identify the most useful fixed algorithms and hierarchies for assigning cause of death in a deterministic manner. For neonates, these include the Compromise hierarchy to be used in high mortality settings and the Baqui hierarchy otherwise; while for 1–59 month–old children, the Kalter hierarchy performed best overall. These expert algorithms provide an accessible and systematic mechanism for interpreting verbal autopsy, on par with more complex machine learning methods that will soon replace the current standard. Work is ongoing to assess the feasibility of mapping the algorithms to the 2014 WHO VA questionnaire in order to render the method even more accessible.
